# Prenatal hypoxia alters the early ontogeny of dopamine neurons

**DOI:** 10.1038/s41398-022-02005-w

**Published:** 2022-06-07

**Authors:** Anastasia Brandon, Xiaoying Cui, Wei Luan, Asad Amanat Ali, Renata Aparecida Nedel Pertile, Suzanne Adele Alexander, Darryl Walter Eyles

**Affiliations:** 1 Queensland Brain Institute, The University of Queensland, St Lucia, QLD Australia; 2 Queensland Centre for Mental Health Research, The Park Centre for Mental Health, Wacol, QLD Australia

**Keywords:** Molecular neuroscience, Psychiatric disorders

## Abstract

Dopaminergic (DA) dysfunction is a significant feature in the pathophysiology of schizophrenia. Established developmental risk factors for schizophrenia such as maternal immune activation (MIA) or developmental vitamin D (DVD) deficiency, when modelled in animals, reveal the differentiation of early DA neurons in foetal brains is delayed suggesting this may be a convergent aetiological pathway. Here we have assessed the effects of prenatal hypoxia, another well-known developmental risk factor for schizophrenia, on developing DA systems. Pregnant mice were exposed to a hypoxic environment of 10% oxygen for 48 h from embryonic day 10 (E10) to E12. Embryonic brains were collected and the positioning of mesencephalic cells, expression of DA specification and maturation factors were examined along with the expression of factors that may govern the migration of these neurons. We show that prenatal hypoxia results in a decrease in dopaminergic progenitors retards early DA neuron lateral migration and reduces expression of the receptors known to govern this process. A second time-point, postnatal day 10 (P10) was also examined in order to assess whether prenatal hypoxia alters early presynaptic architecture in the developing striatum. We show reduced expression of tyrosine hydroxylase (TH) in the postnatal striatum along with increases in the density of high-probability DA release sites within TH varicosities. These findings add to the emerging literature showing that multiple epidemiologically validated environmental risk factors for schizophrenia may induce early alterations to develop DA systems. This may represent a possible convergent mechanism in the onset of presynaptic DA dysfunction in patients.

## Introduction

Schizophrenia affects roughly 1% of the population. Like all complex diseases of unknown cause, the aetiology of schizophrenia is believed to be the sum of an interaction between genetics and environmental factors^1^. Environmental risk factors (RF) that contribute to schizophrenia can occur at any age; however, adverse events during early prenatal development are particularly prominent^2^. Major epidemiologically validated RFs include obstetric complications, maternal immune activation, maternal stress and nutrient deficiencies^3^.

The longest-standing hypothesis in schizophrenia aetiology is that dopaminergic systems are dysfunctional^4^. Blockade of dopamine (DA)2 receptors remains a necessary condition for current antipsychotic action^5^. The best evidence supporting dopaminergic dysfunction in schizophrenia is based on positron emission tomography (PET) studies which indicate patients have increased presynaptic abnormalities in DA uptake, synthesis or release within the dorsal striatum^6^. Subjects who are at a high risk of developing psychosis also have abnormalities in dorsal striatum presynaptic DA uptake and this is most prominent in those that go on to develop schizophrenia^7^. This suggests DA abnormalities may be present well before clinical diagnosis.

When developmental RFs for schizophrenia are modelled in animals they all produce abnormalities in DA release/function in adult offspring. DA neurons appear very early within the mesencephalon, at approximately gestational week 5 in humans^8,9^, and embryonic day (E) 10.5 in mice^10^. Therefore the possibility exists that adverse exposures linked with schizophrenia at these initial stages of pregnancy may actually affect the orderly formation of early DA systems resulting in the abnormalities in DA release and DA-mediated symptoms as adults. Indeed it is intriguing to note that in the few models to date (MIA and DVD deficiency) where foetal brains have been examined proximal to such adverse developmental exposures, early alterations in DA neuron differentiation have been reported. In particular, alterations in DA progenitor cell number, crucial DA specification factors and DA neuron positioning have all been described^11,12^. These findings have prompted us to consider whether early alterations to developing DA systems represent a convergent developmental mechanism in schizophrenia^3,13^.

One of the most longstanding developmental RFs for schizophrenia is obstetric complications which induce pre/perinatal hypoxia^14^. Human autopsy studies have shown that perinatal hypoxia affects the expression of the rate-limiting enzyme in DA synthesis, tyrosine hydroxylase (TH)^15,16^. Several transcription factors that are activated by hypoxic conditions such as SRY (sex-determining region Y)-box 2, (sox2)^17^ regulate the rate of TH expression^18^. Immunohistochemistry conducted on post-mortem samples of 18 human neonates indicates mesencephalic dopaminergic neurons are particularly vulnerable to prolonged perinatal hypoxia^16^. This suggests that like DVD deficiency and MIA, prenatal hypoxia may also adversely affect the orderly formation of DA neurons within the foetal mesencephalon.

Early delays in the differentiation of DA neurons may also lead to abnormalities in connectivity with the striatal target. Catecholamines like DA differ from other neurotransmitters in that most of the DA released in the striatum is extrasynaptic, i.e. meaning that most of the DA released is not in close proximity to a postsynaptic specialised membrane. The DA released still signals via binding to DA receptors both pre- and post-synaptically but the diffusion path is longer than for that seen in a synapse^19,20^. This is referred to as volume transfer. It remains unknown whether the increased striatal release of DA in animal models based on these RFs reflects an increase in active release sites. Therefore we have assessed whether prenatal hypoxia also leads to structural changes in these elements in the early postnatal brain. We achieve this using quantitative immunohistochemistry coupled with quantitative cell recognition software. To assess DA release site density we established the presence of a presynaptic protein (*Bassoon)* inside TH varicosities in the striatum. Bassoon is an active zone scaffold protein that links plasma membrane vesicles with calcium channels indicating a high-probability release site^21^. Bassoon has also been shown to be an essential component of the machinery involved in both synaptic and extrasynaptic DA release^20^.

In this study we investigate the effects of prenatal hypoxia on both early events in DA neuron differentiation within the developing mesencephalon as well as whether this correlates with abnormalities in early postnatal brain DA terminal architecture. Using a machine learning approach we have examined whether prenatal hypoxia affects the expression of DA-relevant proteins, cell number and/or positioning in foetal DA neurons and active release site densities in the postnatal day (PND) 10 striatum. PND 10 represents the earliest stage at which striatal connectivity can be assessed post the last major wave of apoptotic DA cell elimination^22^. Our findings confirm that like other developmental RFs for schizophrenia prenatal hypoxia alters important events in the very early differentiation of developing DA neurons but also leads to increases in presynaptic release sites in the striatum which is consistent with the increased striatal DA release shown in patients. This study confirms the emerging hypothesis that developmental risk factors for schizophrenia may all converge on developing DA systems.

## Materials and methods

### Prenatal hypoxia

C57BL/6 J mice (Animal Resource Centre, Western Australia) were used throughout the study. Animals were housed in a 12 h light/dark cycle (lights on at 07:00 h). Females were group-housed for 2 weeks prior to mating in order to suppress oestrous, then exposed to male bedding 2 days prior to mating to induce oestrous. Females were then time-mated. At E10 dams were either exposed to 10% oxygen (hypoxia) or atmospheric conditions (control 21% O_2_) for 48 h. Dams remained in their home cage which was placed in a special purpose chamber (A-Chamber, Biospherix, 76 × 51 × 51 cm) where gas content was manipulated manually by oxygen and nitrogen infusion (ProOx110, Biospherix). Dams had ad lib access to food and water during the entire procedure and were monitored twice daily. After this treatment all dams were euthanised according to AVMA guidelines^23^, pups retrieved and brains processed for immunohistochemistry

To ensure the embryonic mesencephalon had been subjected to hypoxia, two additional dams, one subjected to hypoxia and one to the control condition were examined with the hypoxia marker, pimonidazole. For images and details of this procedure see Supplementary Fig. 1. As expected prenatal hypoxia adversely affected maternal and pup physiology leading to reductions in maternal weight gain (t = 5.48 *p* < 0.0001) and a very small, (4.1%) but significant reduction in body size (t = 3.46, *p* < 0.001) (Supplementary Fig. 2).

### Effect of prenatal hypoxia on early mesencephalic DA neuron differentiation

#### Tissue collection

The dams exposed to pimidazole were not used for ongoing experiments. This left 8 control and 6 hypoxic dams. At E12, dams were euthanised. Embryos were then quickly removed from the uterus and placed in an ice slurry. From each dam a single embryo was immersion fixed in 4% paraformaldehyde overnight at 4 °C. Fixed embryos were soaked in 30% sucrose solution for 48 h before being embedded in Tissue-Tek O.C.T compound (Sakura, USA). From the remaining embryos the ventral mesencephalon was dissected for later gene-expression studies. Samples were stored at −80 °C until further processing. A complete series of rostrocaudal sections across the mesencephalon was obtained from all six hypoxic embryos but technical difficulties in sectioning meant a complete series could only be obtained from five control embryos. These were all obtained from separate litters. Sex was not determined at this age.

#### Immunohistochemistry

E12 was chosen because at this age, the mesencephalon of mice contains DA neurons at multiple stages of differentiation. Sox2 is a neural stem cell marker. Sox2 actively suppresses the expression of differentiation factors therefore its presence indicates an undifferentiated cell^24^. Lmx1a is the gene that encodes the LIM homeobox transcription factor 1, alpha protein. Lmx1a is an early differentiation marker for mesencephalic (ms) DA neurons and is essential for the specification, proliferation and differentiation of dopaminergic progenitors^25^. Therefore the co-location of Lmx1a and Sox2 in the ventral mesencephalon represents likely DA progenitors. When Sox2 expression is switched off in these neurons this represents a very early stage in the differentiation of these progenitors making (Sox2− Lmx1A+) cells likely immature DA neurons. The presence of the rate-limiting enzyme in dopamine synthesis, tyrosine hydroxylase (TH) reflects a postmitotic differentiated DA neuron. These more mature dopaminergic neurons expressing TH are found in the mantle zone before migrating both radially and tangentially to form the ventral tegmental area (VTA) and substantia nigra (SN).

Embryonic mesencephalon coronal sections (20 μm) were obtained in a one in six series using a cryostat. One series were selected for Sox2/Lmx1a staining and another series for TH. All sections from each experimental group were processed at the same time in order to exclude batch variation for the quantitative analysis of protein expression. Sections were rinsed in phosphate-buffered saline (PBS) and blocked for 4–6 h in 10% normal donkey serum (NDS) in PBS with 0.1%Triton X-100 (PBST). The sections were then incubated with the primary antibodies diluted in blocking solution overnight at room temperature. The primary antibodies included rabbit anti-Sox2 (1:200, Millipore, Australia), goat anti-Lmx1a (1:100, Santa Cruz, USA), and sheep anti-TH (1:100, Novus Biology, Australia). After four washes with PBST (15 min each), the sections were incubated with fluorophore-conjugated secondary antibodies diluted in a blocking solution, for 4–6 h at room temperature. The following secondary antibodies were used: Alexa-555 conjugated donkey anti-goat (1:1000, Thermo Fisher Scientific, USA), Alexa-647 conjugated donkey anti-rabbit (1:1000, Thermo Fisher Scientific, USA) and Alexa-488 conjugated donkey anti-sheep (1:1000, Thermo Fisher Scientific, USA). Cell nuclei on all sections were visualised with 4’,6-diamidino-2- phenylindole (DAPI, 1:100, Sigma–Aldrich, Australia). Sections were washed again four times for 15 mins and cover-slipped using Dako Fluorescence Mounting Medium (Dako, USA).

#### Microscopy and quantitative analysis

All images were acquired using a Diskovery™ spinning disk module with an inverted spinning disk. Images of the entire midbrain for E12 embryos were captured using a ×60 oil N.A. 1.4 objective (CFI Apo Lamda /W.D. 0.14 mm), with a fixed exposure time for each channel with a pixel size 0.092 µm^2^ using NIS software (Nikon Inc. USA). The images were processed using ImageJ software (version 1.51, National Institutes of Health, USA) and subsequently analysed with CellProfiler software (version 3.0.0, Broad Institute, USA) as previously described^12^. More details of this analysis pipeline and descriptions of how cell number, protein intensity, mediolateral and mediodorsal positioning were obtained are described in Supplementary methods and images detailing this analysis pipeline are described in Supplementary Fig. 4.

To verify the detection accuracy and correct segmentation of the CellProfiler pipeline (the percentage of correctly identified and segmented cells according to the human observer), msDA cell types were counted manually by blinded researchers in randomly selected regions (500 × 500 pixels) in the region of interest (ROI) and compared to the automated segmentation. The accuracy of our analysis pipeline in identifying msDA progenitors (Lmx1a+/Sox2+), likely immature DA neurons (Sox2− Lmx1a+) and early mature DA neurons (TH+) was 82.64 ± 9.6%; 92.85 ± 1.8% and 93.92 ± 6.8%, respectively.

#### Quantitative PCR (qPCR)

msDA lateral migration is governed by L1 cell adhesion molecule (LICAM) and its receptor, tyrosine phosphatase receptor type Z polypeptide 1 (PTPRZ1)^26^ and Reelin and its receptor disabled 1 (dab1)^10^ To examine the expression of these genes ventral midbrains were dissected from 2 embryos/litter and kept at −80 °C until use. Each sample was homogenised with 600 μl of Trizol (Thermo Fisher Scientific) and then 200 μl of chloroform. The samples were centrifuged at 12,000 × *g* at 4 °C for 15 min, and the upper aqueous phase recovered. One volume of 70% ethanol was added to each tube and the samples were transferred to the RNeasy Spin column (Qiagen), and RNA extracted using RNAeasy Micro Kit (Qiagen). cDNA synthesis was performed using the SensiFAST™ cDNA synthesis kit (Bioline, London, United Kingdom). qPCR reactions were prepared with the SensiFAST™ SYBR® master mix (Bioline, London, United Kingdom). Primer sequences and thermal cycling conditions are provided in Table 1 in the Supplementary materials.

### Effect of prenatal hypoxia on striatal DA terminals

#### Animals

Six hypoxic and eight normoxic dams were allowed to litter normally. At PND 10, one single pup from each litter was intra-cardially perfusion-fixed using 4% paraformaldehyde (PFA) prior to obtaining brains. Brains were post-fixed in 4% PFA overnight before being transferred into 1×PBS with 0.01% sodium azide until paraffin processing.

#### Tissue processing and immunohistochemistry

Fixed paraffin brains were sectioned at 10 µm thickness using a Rotary Microtome Leica RM2235 and mounted on Uberfrost glass slides and dried overnight at ~40 °C. Deparaffinisation was conducted at 60 °C for 30 min. Slides were then cleared and rehydrated by being placed in xylene for 2 × 15 min and 100% ethanol for 2 × 5 min, followed by 70% ethanol for 3 min, then tap water. Slides were then placed into an antigen recovery solution and placed in a decloaking chamber at 95 °C for 10 min. Slides were then washed with 1× PBS (10 min × 3 times), and blocked for 4–6 h at room temperature with 0.05% sodium azide, 0.1% Triton X-100. Primary antibodies including rabbit anti-Bassoon (1:200, Synaptic System, Australia) and sheep anti-TH (1:200, Novus Bio, USA) were diluted in a blocking solution and incubated overnight at room temperature. For each antigen all experimental sections were processed at the same time. After four washes with PBST (15 min each), the sections were incubated with fluorophore-conjugated secondary antibodies diluted in a blocking solution, for 4–6 h at room temperature. The following secondary antibodies were used: Alexa-647 conjugated donkey anti-rabbit (1:1000, Thermo Fisher Scientific, USA) and Alexa-488 conjugated donkey anti-sheep (1:1000, Thermo Fisher Scientific, USA). Nuclei were stained with 4’,6-diamidino-2- phenylindole (DAPI, 1:100, Sigma–Aldrich, Australia). Sections were washed again four times for 15 min and cover-slipped using Dako Fluorescence Mounting Medium (Dako, USA).

#### Microscopy and quantitative analysis

A single section containing both the dorsal and ventral (Nucleus Accumbens, NAc) striatum was used to examine DA terminals unilaterally. The position chosen corresponded to approximate bregma 0.74 vmm in an adult mouse brain. A site 1 mm below the olfactory tubercule within the NAc shell was chosen as this represents a clear projection target of DA neurons within the VTA with no overlap from projections from DA neurons within the SN. A site 1 mm medial from the most lateral point of the striatum was selected as the region within the dorsal striatum to be examined as this represents projections of DA neurons from the SN with little overlap from DA neuron projections from the VTA. The mean of five non-overlapping images (128 × 128 µm) (centre, top, bottom, left and right) of each of the two regions was scanned using a Zeiss Axio Observer Z1 equipped with a W1 spinning disk (Yokogawa) under a ×100 objective lens (1.4 NA) (refer to Supplementary Fig. 4). Huygen Professional software (Scientific Volume Imaging, Netherlands) was used to deconvolve images. Deconvolved TIFF images were then analysed using Imaris software (Bitplane, version 9.4). High-probability axonal DA release sites were identified as Bassoon+ spots within TH varicosities^19,20^. One NAc sample was damaged during tissue processing and so could not be assessed.

### Statistical analysis

Mesencephalic DA neurons were analysed by multivariate analysis of variance to determine the main effect of group (hypoxia and control), the main effect of position and group × section position interaction on cell number, protein expression and mediolateral and dorsoventral position. Where interaction between group × section position was found this was followed by post-hoc tests. Gene expression was assessed by *t*-test with Bonferroni-Dunn corrections for multiple comparisons.

For measures of early striatal DA connectivity the mean values of the five images per brain region from a single section represented the mean value for that animal. Means from all animals were analysed by analysis of variance to determine the main effect of group (hypoxia and control), on TH axon number, TH axon area, TH protein expression and the number of activated release sites. Statistical significance was defined as *p* < 0.05. All statistical analyses were performed using SPSS software (version 23, SPSS Inc.).

## Results

### Effect of prenatal hypoxia on early mesencephalic DA neuron differentiation

#### Dopaminergic progenitors: (Lmx1a+/Sox2+)

**Fig. 1:**
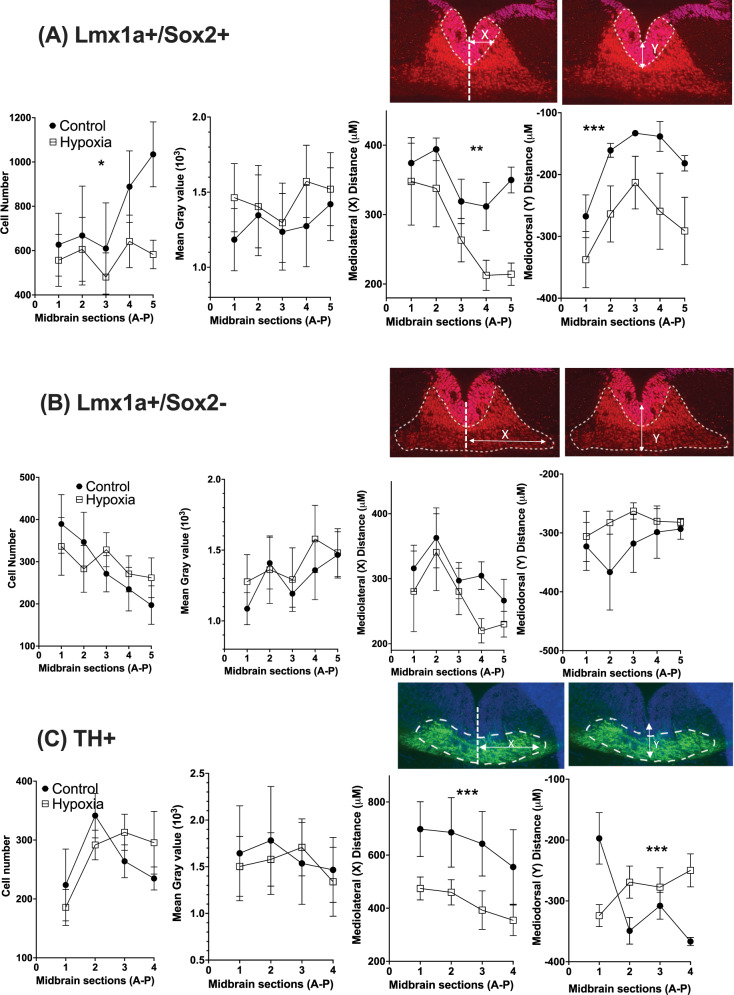
Prenatal hypoxia alters the early ontogeny of DA neurons. **A** Dopaminergic (DA) progenitors (Lmx1a+/Sox2+). There was a reduction in DA progenitor number (Lmx1a+/Sox2+); no difference in Lmx1a expression (mean Grey value); a reduction in DA progenitor mediolateral (X) positioning (relative to midline); and an increase in DA progenitor dorsoventral positioning (Y) (from the ventral boundary of the mesencephalic aqueduct) in E12 hypoxic brains. Image shows brain region examined. A anterior, P posterior. (Control, *n* = 5; Hypoxia, *n* = 6; data are shown as mean ± SEM; Group differences are indicated *p < 0.05, **p < 0.01, ***P < 0.001). **B** Postmitotic (Lmx1a+/Sox2-) cells Hypoxia had no effect on the number of postmitotic (Lmx1a+/Sox2−) cells; no effect on Lmx1a expression (mean Gray value); no effect on mediolateral lateral (X), positioning (relative to midline); and no effect on dorsoventral positioning (Y) (from the ventral boundary of the mesencephalic aqueduct) in E12 hypoxic brains. Image shows brain region examined. A anterior, P posterior. (Control, *n* = 5; Hypoxia, *n* = 6; data are shown as mean ± SEM). **C** Differentiated or mature dopaminergic neurons (TH+) Hypoxia had no effect on the number of mature dopaminergic neurons (TH+); no effect on TH expression; however, there was a reduction in mediolateral (X), positioning (relative to midline); but no alteration in dorsoventral positioning (Y) (from the ventral boundary of the mesencephalic aqueduct) in E12 hypoxic brains. There was also a group/section interaction where the rostrocaudal reduction in TH+ neurons seen in controls was absent in hypoxic brains. Image shows brain region examined. A anterior, P posterior. (Control, *n* = 4, Hypoxia, *n* = 6; data are shown as mean ± SEM; Group differences are indicated ***P < 0.001).

There were significant effects of treatment but no significant interactions between treatment and section position, therefore only treatment effects are reported. There was a significant effect of maternal hypoxia on DA progenitor cell number. DA progenitor cell number was significantly reduced in hypoxic brains (F_1, 55_ = 4.41, *p* = 0.041). There was no effect of hypoxia on Lmx1a protein expression in progenitor cells (F_1, 55_ = 0.99, *p* = 0.32). Mediolateral positioning of DA progenitors from the midline was significantly reduced in hypoxic brains (F_1, 55_ = 10.20, *p* = 0.003); however, their dorsoventral positioning migration from the midline was significantly increased in hypoxic brains when compared to controls (F_1, 55_ = 13.59, *p* = 0.001) (Fig. 1A).

#### Postmitotic DA neurons: (Lmx1a+/Sox2−)

There was no observed effect of hypoxia on postmitotic dopaminergic cell number (F_1, 55_ = 0.06, *p* = 0.81), Lmx1a expression (F_1, 55_ = 0.59, *p* = 0.45), nor was there an effect of hypoxia on mediolateral (F_1, 55_ = 1.29, *p* = 0.27) or dorsoventral positioning of these neurons (F_1, 55_ = 2.37, *p* = 0.14) (Fig. 1B).

#### Early mature dopaminergic neurons: (TH+)

There were significant effects of treatment and significant interactions between treatment and section position for early mature TH-positive neurons. There was no significant effect of hypoxia on TH cell number in early mature dopaminergic neurons (F_1, 40_ = 0.04, *p* = 0.84). There was also no effect of hypoxia on TH protein expression (F_1, 40_ = 0.07, *p* = 0.79). There was an effect of hypoxia on TH-neuron mediolateral positioning, whereby hypoxic TH neurons failed to migrate as far laterally as controls (F_1, 40_ = 13.77, *p* < 0.0001). There was no effect of hypoxia on TH dorsoventral positioning (F_1, 40_ = 1.65, *p* = 0.21). However, there was a significant interaction between dorsoventral position and anterior-posterior positioning whereby TH-neuron ventral position increased caudally in hypoxic brains but the reverse was the case in controls (F_1, 40_ = 7.55, *p* < 0.001) (Fig. 1C).

#### Gene expression in the E12 ventral mesencephalon

**Fig. 2:**
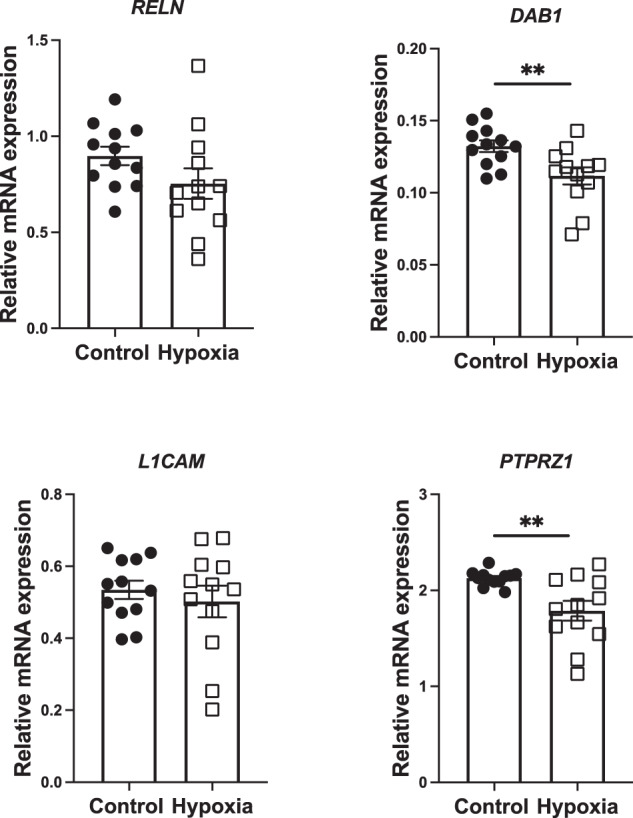
Prenatal hypoxia reduces the expression of receptors governing lateral DA neuron migration of early DA neurons. Hypoxia reduced the expression of the receptors DAB1 and PTPRZ1. However, hypoxia had no effect on the expression of their respective ligands Reelin and L1CAM. (Control, *n* = 12, Hypoxia, *n* = 12; data are shown as mean ± SEM; Group differences are indicated **P < 0.01).

To measure whether the altered positioning of DA cells was due to alterations in the expression of molecular cues for cell migration, we quantified the expression of the genes for two prominent ligands and their respective receptors which are involved in the lateral migration of DA neurons in the embryonic mesencephalon. Although we saw no change in either ligand (LICAM and Reelin) we did see a reduction in the expression of both their receptors PTPRZ1; (mean difference = 0.34, *t* = 3.2, df = 22 *p* = 0.01) and dab1 (mean difference = 0.02, *t* = 2.9, df = 22 *p* = 0.03) (Fig. 2).

### Early dopaminergic terminal architecture in the PND 10 dorsal and ventral striatum

**Fig. 3:**
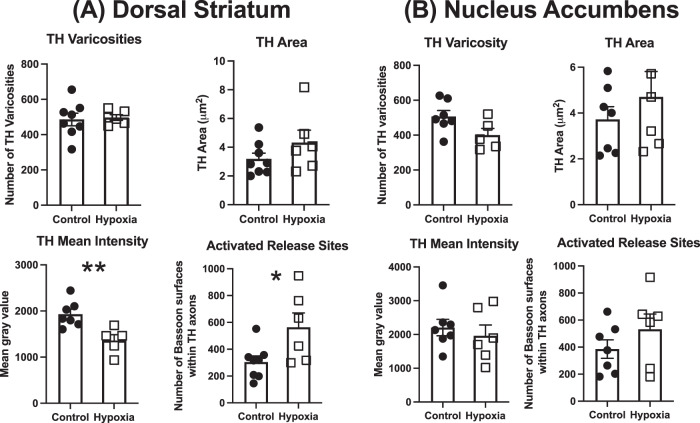
Prenatal hypoxia alters DA terminal architecture in the P10 dorsal striatum. **A** There was no effect of prenatal hypoxia on TH varicosity number or area covered by TH varicosities in P10 dorsal striatum. Prenatal hypoxia led to a significant reduction in the intensity of TH expression and increased the number of activated release sites. **B** There was no effect of prenatal hypoxia on TH varicosity number, area covered by TH varicosities, intensity of TH staining or activated release site number in P10 nucleus accumbens. (Control, *n* = 8, hypoxia, *n* = 6; data are shown as mean ± SEM; Group differences are indicated by *p < 0.05, **p < 0.01).

In the dorsal striatum there was no effect of prenatal hypoxia on the number of TH varicosities (F_1, 11_ = 0.05, *p* = 0.83), or the percentage area covered by TH surfaces across the regions sampled (F_1, 12_ = 1.67, *p* = 0.22). However, prenatal hypoxia was found to significantly reduce the intensity of TH protein expression within axonal surfaces (F_1, 10_ = 11.93, *p* = 0.006). Additionally, hypoxia significantly increased the number of high-probability DA release sites (F_1, 12_ = 6.08, *p* = 0.03) (Fig. 3A). In contrast there was no significant effect of prenatal hypoxia on any of the same parameters within the shell of the NAc with no change in the number of TH varicosities (F_1, 10_ = 4.31, *p* = 0.065), axonal area covered by TH surfaces (F_1, 11_ = 0.68, *p* = 0.43), intensity of TH expression (F_1, 11_ = 0.38, *p* = 0.55), number of non-synaptic release sites (F_1, 11_ = 1.33, *p* = 0.27) (Fig. 3B).

## Discussion

After 48 hrs of maternal hypoxia there are immediate changes in developing msDAs. There are fewer DA progenitors formed by E12 and they migrate further ventrally and less laterally compared to control brains. Immature msDA neurons that have immediately exited the cell cycle appear normal; however, there are implications for early differentiated TH-positive neurons. Although there are no fewer TH-positive neurons, like the progenitors their lateral migration again is retarded. Collectively these findings imply prenatal hypoxia leads to impairments in the orderly formation of the very early mesencephalon. In the postnatal brain, prenatal hypoxia induces alterations in dorsal striatal connectivity with a reduction in overall intensity of TH staining within these early axons and an increase in high-probability release sites within TH varisocities. In summary maternal hypoxia at this early gestational period would appear to affect the orderly differentiation and positioning of newly born msDA neurons leading to abnormal terminal architecture in the early striatum.

To the best of our knowledge, in vivo studies on the effects of hypoxia on msDA neurons at earlier stages of development (E10 –E12) have not been reported. However, with respect to DA progenitor formation our results are at least congruent with the only study to date that has examined varying oxygen concentrations on developing msDA neurons in vivo^27^ When C57Bl6 mouse dams were exposed to 48 h of hypoxia (10% O_2_) from E14 to E16, msDA neuron number was reduced when assessed at E16^27^. Interestingly this reduction was again greater laterally in an area that will form the SN compared to medial neurons that will form the VTA. This is important given we find here prenatal hypoxia reduces early (E12) lateral migration of TH neurons along with reductions in expression of guidance molecules for this migration (see below). The Wagenfuhr et al., study also examined prenatal hyperoxia (75% O_2_) at the same age examined here from E10 to E12. The findings are reversibly consistent with our data in these authors showed hyperoxia increases TH cell number^27^.

Hypoxia-induced reductions in progenitor number in vivo are in stark contrast to studies that induce hypoxia in primary msDA neurons in culture. Most such studies show low oxygen increases DA progenitor formation^28–30^. However, these studies are conducted on cultures that are made from embryonic neurons that are largely postmitotic i.e. E14. It is possible that the pro-differentiation mechanisms that hypoxia acts on in vitro do not yet exist at the earlier embryonic ages that were examined in our study. Physiological responses to prenatal hypoxia are mediated by the powerful transcription regulator Hypoxia-inducible factor 1-*alpha*, which is well known to induce a variety of proapoptototic proteins^31^. However, increased apoptosis is unlikely to be the explanation for the reduction in progenitors at such an early developmental age. Clearly, there are different factors mediating the effects of oxygen on DA neuron outcomes depending on when and how they are assessed.

We also show the early lateral positioning of DA progenitors and early mature DA neurons in hypoxic brains was retarded. Neurons destined for the SN migrate radially and then laterally and neurons destined for the VTA primarily migrate radially^10^. msDA neurons that migrate laterally to form the SN migrate along tangentially oriented fibres that express the neural cell adhesion molecule L1CAM (L1 cell adhesion molecule). Migrating msDA neurons express the L1CAM receptor protein tyrosine phosphatase, receptor type Z, polypeptide 1 (PTPRZ1)^26^. Inactivating L1CAM blocks this migration^32^. A second major molecule governing lateral msDA migration is Reelin. The Reelin receptor, disabled 1 (dab1), is restricted to the most lateral SN DA neurons that form the *pars compacta*, (SNpc)^10^. Genetically ablating Reelin or dab1 blocks the formation of the SNpc with mDA neurons accumulating in the VTA^10,33–35^. When Reelin expression is reduced in primary cultures the speed and trajectory of tangential but not radial migrating DA neurons are affected^10^. Our findings of a reduction in both receptors for these lateral guidance molecules are highly suggestive of a plausible mechanism for this initial delay in lateral positioning of TH neurons.

A delay or a reduction in lateral migration of mDA progenitors is consistent with delayed differentiation of these progenitors. Additionally, the finding that ventral migration appears accelerated possibly means that the lateral signalling cues were not observed. A reduction in lateral positioning of both progenitors and early mature TH neurons within the mesencephalon implies prenatal hypoxia may impair the orderly formation of the SN. We also identified a significant interaction between dorsoventral position and anterior-posterior sections, for early TH neurons; however, the molecular cues responsible for how rostrocaudal DA neurons migrate remain largely unexplored^36^. Whether these early reductions in the lateral migration of DA progenitors and postmitotic DA neurons represent a delay in SN DA neuron maturation or a permanent alteration can not be deduced from a single time-point and would require a developmental time-course study. In any case, either outcome could lead to persistent regional alterations in dopaminergic innervation of the dorsal striatum producing variable symptom outcomes of relevance to schizophrenia^37^.

Finally, we considered how the well-known reduction in foetal size induced by prenatal hypoxia may compromise the spatial interpretation of cell migration data. However, we consider this unlikely to be a significant confound in our study for several reasons. 1st the reduction in embryo size observed here is extremely small, 4.1%; 2nd some spatial findings were even increased in hypoxic brains i.e. progenitor ventral migration; 3rd a reduction in embryo brain size can not explain the rostrocaudal dorsoventral interaction found in TH+ cells between control and hypoxic brains.

Prenatal hypoxia is known to produce long-lasting deleterious effects on synaptogenesis and connectivity within the postnatal brain^38^. Therefore the next question we addressed was whether the early alterations in msDA neuron differentiation found here would also affect DA terminal architecture in the early postnatal striatum? To examine this we repeated the prenatal exposure but allowed pups to be born and we examined the striatum at PND 10. Although DA afferents begin to reach the striatum much earlier than this, this time-point represents an initial point of stability in the early innervation of DA axons into the striatum being just posted the 1st major wave of DA neuron elimination by apoptosis^22^.

We show that although the overall number and coverage of TH varicosities were unaltered, TH expression in the dorsal striatum was reduced. This may indicate a persistent effect of delayed DA neuron differentiation/lateral migration in forming the SN. Somewhat paradoxically, there was an increase in both the number of activated release sites within TH varicosities from this same region. Most DA released in the striatum is primarily via volume transfer i.e. non-synaptic^19,20,39^. Therefore our finding of increased DA release site density indicates that DA terminal architecture is altered in a manner consistent with the heightened DA release observed in adult offspring subjected to pre- or perinatal hypoxia^40,41^.

The enhanced number of DA release sites seen here in postnatal brains that are still developing could possibly represent a form of sprouting. In models of Parkinson’s disease when substantial reductions in SN cell number are induced via 6-OHDA lesions, this induces sprouting^42^. DA terminals formed by sprouting have increased the number of vesicles, increased terminal bouton size and contacts to more proximal targets^43^. Importantly the increases in TH variscosities and TH-specific boutons produced in these lesion models occur alongside a reduction in axonal TH content^44^ representing a similar scenario to that found in the dorsal striatal tissue from this study.

If these findings are not compensated for at later stages of development then we predict that the mature dorsal striatum of mice subjected to prenatal hypoxia will possess increased DA release potential but with diminished synthesis capacity. As previously explained, the literature from preclinical models of Parkinson’s disease may indicate that a reduced DA cell number and therefore capacity to synthesise DA is partially compensated by an increase in presynaptic release sites. This makes it difficult to predict the response of the dorsal striatum in such animals to phasic stimuli such as stress or DA-releasing drugs such as amphetamine. Initial rates of DA release may be increased but a reduced synthetic capacity may truncate any initial increase and may even retard overall DA response. The short and long-term profiles of DA release within the mature dorsal striatum subjected to prenatal hypoxia exposure using techniques such as fast-scan cyclic voltammetry or dialysis, respectively, are now required.

Striatum-specific changes in DA signalling are consistent with the most up to date literature concerning DA abnormalities in schizophrenia. PET studies within patients with schizophrenia clearly show that DA uptake /synthesis and release abnormalities are across the striatum but are most prominent in the dorsal striatum^6^. Given prenatal hypoxia is a well-known epidemiological risk factor for schizophrenia our findings may indeed represent one important late-developmental mechanism. However, although no significant effects of prenatal hypoxia on DA terminals were reported in the NAc, the directionality of all findings was similar to that shown in the dorsal striatum perhaps indicating our study was underpowered to find such effects.

Pre- and perinatal hypoxia are both risk factors for schizophrenia; however, our findings here are of more relevance to early prenatal hypoxia. Often women may be unaware that they are pregnant during their 1st trimester and may continue hypoxia-inducing behaviours such as smoking during this early prenatal period. The 1st trimester corresponds to the period where all DA neurons are born in the human foetus (refs. ^8,9^). E10–E12 in the mouse roughly corresponds to a period where virtually all DA neurons are born in the mouse. We felt it was important therefore to model this very early period of prenatal hypoxia. The effects of perinatal hypoxia on DA neuron ontogeny have been poorly explored.

### Is there consistency between RFs?

Finally, these findings must be considered within the context of recent data indicating other prenatal risk factors for schizophrenia also affect the early ontogeny of DA neurons^3,13^. DVD deficiency^45,46^ and MIA^2,47^ are known developmental risk factors for schizophrenia. The animal models based on these risk factors as well as hypoxia produce convincing behavioural phenotypes of relevance to schizophrenia and increase striatal DA release in adult offspring (for a recent review see ref. ^3^. In particular, models of MIA^12^ and DVD deficiency^11^ also suggest medio/lateral migration of embryonic msDA neurons is reduced. Therefore it would appear that these epidemiologically validated developmental RFs for schizophrenia and now with the data presented here for hypoxia, all induce early delays in the formation of the SN. This may prove mechanistically relevant to the altered DA release profile observed in schizophrenia which consistently appears to be in the projection terminals of SN DA neurons, the dorsal striatum^6^.

## Conclusion

**Fig. 4:**
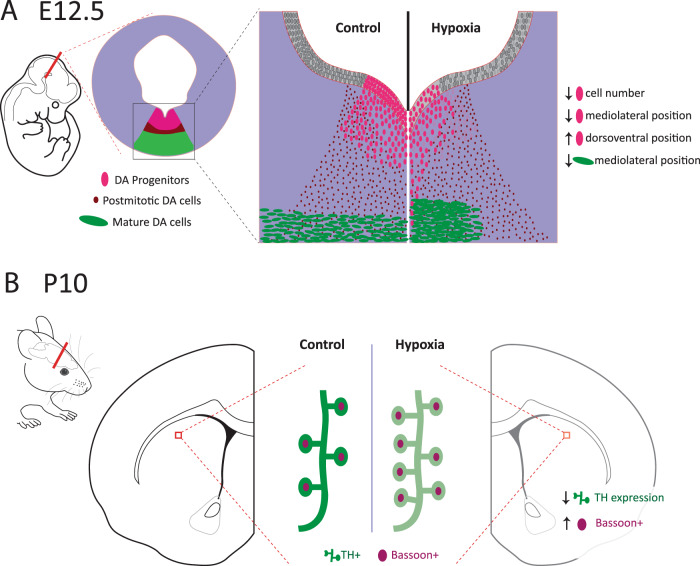
Summary figure of the effects of prenatal hypoxia on dopamine neuron ontogeny. **A** Prenatal hypoxia induces early changes progenitor cell number, progenitor lateral and ventral positioning and a reduction in lateral positioning of more mature TH+ neurons. **B** This correlates with a postnatal reduction in the intensity of TH expression and a potentially compensatory increase in presynaptic release sites in TH-positive terminals. Importantly these findings are in the dorsal striatum not the nucleus accumbens consistent with the anatomical loci of presynaptic DA abnormalities reported in schizophrenia.

Here we have outlined both the proximal effects of prenatal hypoxia on the early development of DA neurons as well as the postnatal implications of prenatal hypoxia on early DA terminal architecture and connectivity in the striatum. For a summary of these findings please see Fig. 4. Delays in early DA neuron progenitor formation and alterations to both progenitor and early mature DA neuron migration indicate a delay in the maturation processes of these neurons. The findings of this study contribute to the emerging concept that multiple developmental risk factors for schizophrenia may be operating to disrupt the orderly development and differentiation of early DA neurons^3,13^. In short these findings support the concept that early alterations to the ontogeny of DA systems may be a convergent mechanistic pathway to abnormal DA function in the adult and by inference, in the onset of schizophrenia.

## Supplementary information

suppl methods and data
