# Supplementary material for: Prenatal hypoxia alters the early ontogeny of dopamine neurons
Source: Transl Psychiatry. 2022 Jun 7;12:238. doi: 10.1038/s41398-022-02005-w (PMC9174174; doi:10.1038/s41398-022-02005-w)
Supplement: Supplementary file 1 — suppl methods and data [file 41398_2022_2005_MOESM1_ESM.docx]

**Ensuring embryonic midbrain hypoxia**

Hypoxic cells bind 2-nitroimidazoles to peptide thiols, where oxygen competes for the addition of the first electron to pimonidazole accounting for the oxygen dependence of binding (Varghese A. Biochem Biophys Res Commun. 1983 16;112(3):1013-20). This binding occurs only in cells that have a partial pressure pO_2_ of <10 mm Hg (hypoxic) and results in irreversible and stable adducts that can be identified using immunohistochemistry.

As outlined in the methods after 47 hrs of hypoxia, one additional hypoxia exposed dam and one control dam (not exposed to hypoxia) were injected with pimonidazole HCl 60 mg/kg body weight, (ip) in a volume of 1ml/kg, returned to their respective chambers for 1 hr then were euthenased and embryos recovered and drop-fixed in 4% paraformaldehyde and frozen 20μm sections obtained according to our stated methods. Mesencephalic coronal and sagittal sections were then thawed and fixed in cold acetone (4°C) for 10 minutes. The sections were rinsed and incubated overnight at 4°C with mouse monoclonal anti-pimonidazole antibody (clone 4.3.11.3) (Mab1) diluted in PBS containing 0.1% bovine serum albumin and 0.1% Tween 20. The sections were then incubated for 90 minutes with Cy-3-conjugated goat anti-mouse antibody 1:150 (Jackson Immuno Research Laboratories). Between all steps the sections were rinsed with PBS three times for 2 minutes. Sections were cover-slipped using Dako Fluorescence Mounting Medium (Dako, USA).

Images were acquired using a Diskovery™ spinning disk module with an inverted spinning disk. Images of the entire midbrain for E12 embryos were captured using a 20x N.A. 1.4 objective (CFI Apo Lamda /W.D. 0.14mm), with an exposure time of 700ms. Visual assessment of pimonidazole HCl immunochemical staining in control and hypoxic tissue indicated a clear increase in staining in hypoxic tissue in both the ventral mesencephalon and the entire E12 embryo **(Suppl Fig 1).**

**Suppl Figure 1:** Hypoxyprobe staining of control (left) and hypoxic (right) embryonic brains clearly showing hypoxia across the brain including the ventral mesencephalon.


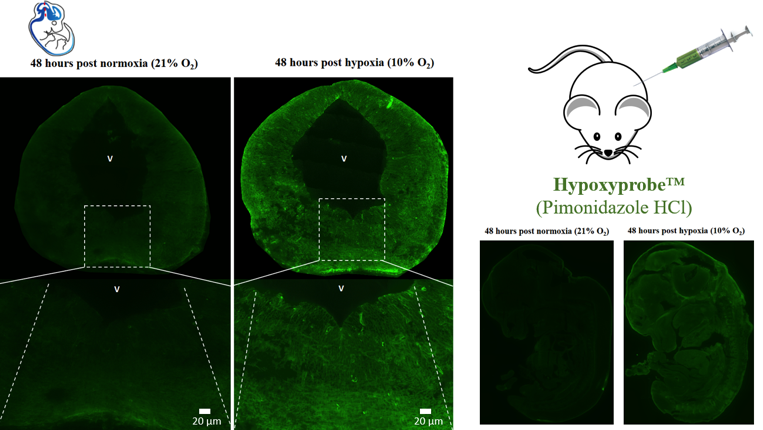


**Effect of hypoxia on maternal weight gain**

We also assessed the general physiology of both dams and foetuses after hypoxia. Seven dams subjected to hypoxia and nine dams subjected to normoxia were weighed both immediately prior to exposure and at the end of exposure. Normoxic dams continued to gain weight during pregnancy as expected but hypoxic dams actually lost weight (t = 5.48 p <0.0001) (**Suppl Fig 2**).

**Suppl Figure 2:** There was a significant effect of maternal hypoxia on normal weight gain during gestation. As expected, control dams (filled circles) as a group gained weight from E10 to E12 whereas hypoxic dams (open squares) actually lost weight (t = 5.48 p <0.0001)

**Effect of hypoxia on maternal weight and foetal growth**

A reduction in maternal weight after hypoxia suggested that there may also be a corresponding reduction in foetal growth. We therefore also measured crown-rump length of all resultant embryos at the E12 time point.

**Suppl Fig 3:** Crown rump length of embryos at E12. Consistent with weight loss in hypoxic dams across 2 days of hypoxia during early gestation, there was a small (4.1%) but significant reduction in embryo size as assessed by measuring crown rump length of embryos at E12 (t=3.46, p < 0.001)

## **Microscopy and Quantitative Analysis details**

Briefly, background fluorescence of images were subtracted using rolling ball method, following removal of outliers and local contrast enhancement. The processed channel and original channel files were analysed using a CellProfiler® pipeline as previously reported for dopamine cell identification (Luan et al; Sci reports 2018: 8 (1): 9741). Accuracy was calculated as the number of correctly identified and rejected objects (true positive x 100 / true positive) + false negative + false positive + over segmented objects + under segmented objects. Quantification of the protein expression of Lmx1a, Sox2 and TH was recorded as the mean florescence intensity/cell. The pipeline also returns other measurements including medio-lateral (x) and dorso-ventral (y) cell position. Medio-lateral and dorso-ventral positioning of individual cells was determined relative to the most ventral point of the ventricle along the midline coordinates for each section (x0, y0). The mediolateral positioning (x) of mesDA cells was measured bilaterally as the absolute distance from the centre of mesDA nucleus (x_n_) to the coordinate (x_0_) (x_0_ – x_n_). Similarly, the dorsoventral positioning (y) of mesDA cell was calculated as the distance from the centre of mesDA nucleus (y_n_) to the coordinate (y_0_) = (y_0_ – y_n_) and the mean value from each cell reported.


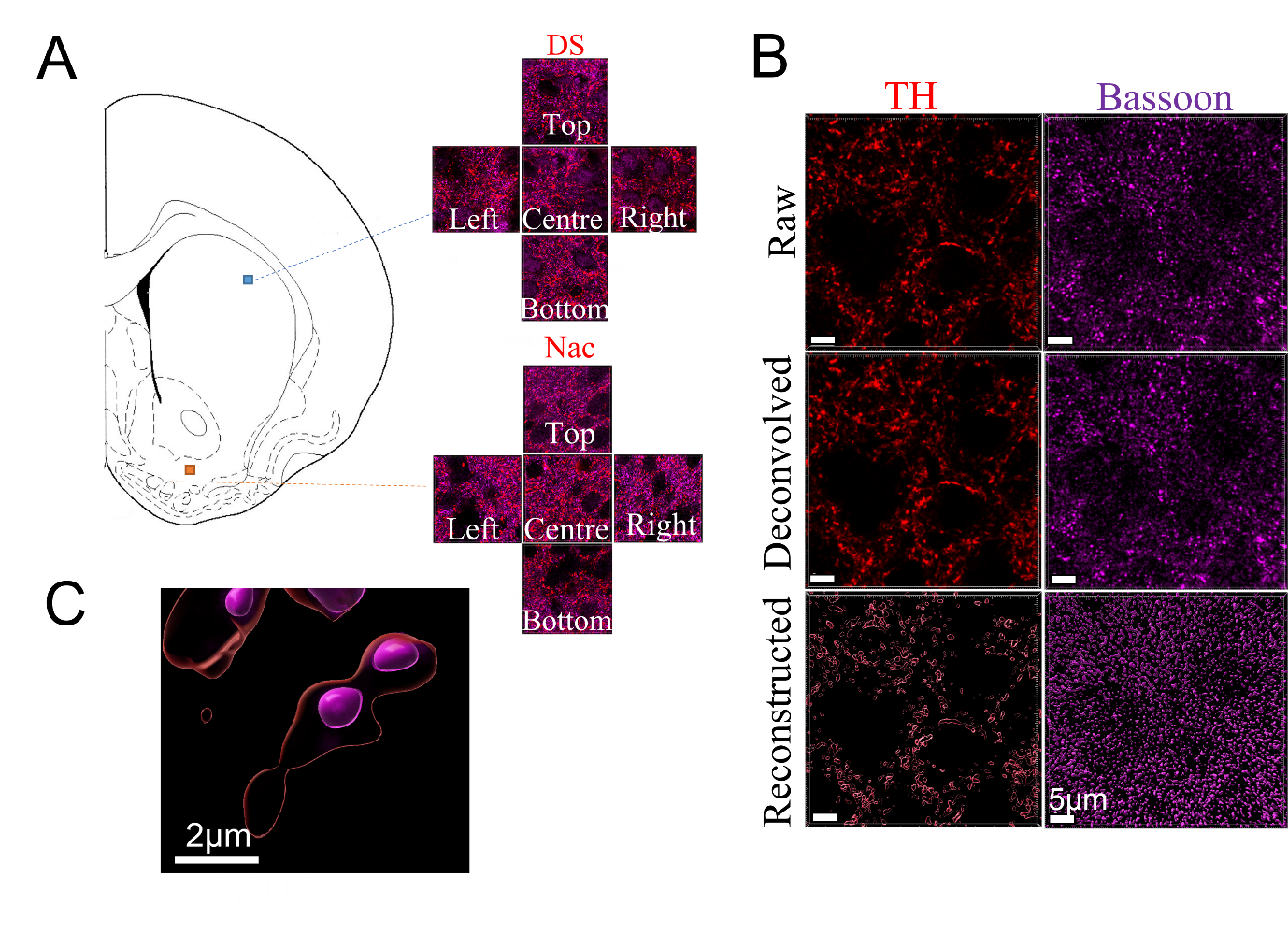


**Suppl Fig 4:**

**Suppl Fig 4A:** Showing sagittal position for estimation of DA release site and synapse number in Nucleus accumbens (NAc) shell and dorsal striatum (DS). The mean of 5 sites as represented was used for each embryo. **B:** Deconvolution and Imaris reconstructed identification of TH axon surfaces (red) and bassoon surfaces (magenta). **C:** Imaris reconstruction of a high probability release site for DA represented as bassoon surfaces within TH variscosities.

**qPCR Conditions and primers used**

qPCR was performed on a LightCycler 480 system (Roche, Basel, Switzerland) and the thermal cycling conditions are listed as follows: a denaturation step at 95 °C for 2 min and then amplification for 40 cycles (95 °C for 5 s, 62 °C for 10 s, and 72 °C for 20 s). Primer sequences are supplied below in suppl Table 1. The relative expression of all genes was normalized to Hypoxanthine-Guanine Phosphoribosyltransferase (HPRT) as the housekeeping gene and the results were analysed using the comparative threshold method.

**Suppl Table 1**. qPCR primer sequences

| Gene | Primers |
| --- | --- |
| *RELN* | Forward 5’- GCTTAGGCGATACCCATGAAG -3’  Reverse 5’- CGCACTGACGTGAAGACATT -3’ |
| *DAB1* | Forward 5’- TCTGTTGAACGCAGAACCTG -3’  Reverse 5’- GGTCTGAAGCAAGGGTACGA -3’ |
| *L1CAM* | Forward 5’- TTGAGTAACGTGCAGCCAAG -3’  Reverse 5’- TCGTTCATCCTGAAGCACTG -3’ |
| *PTPRZ1* | Forward 5’- AGGAGACACTTCCCATGTGC -3’  Reverse 5’- ACTGCCCTCTTTAGGCAACA -3’ |
| *HPRT* | Forward 5’- CAGTACAGCCCCAAAATGGT-3’  Reverse 5’- TTGCGCTCATCTTAGGCTTT-3’ |
